# Communication between patients and primary care physicians after behavioural weight loss: an observational study

**DOI:** 10.1017/S1463423619000124

**Published:** 2019-06-25

**Authors:** Jocelyn E. Remmert, Adam G. Tsai, Savannah R. Roberts, Meghan L. Butryn

**Affiliations:** 1 Center for Weight, Eating, and Lifestyle Science, Department of Psychology, Drexel University, Philadelphia, USA; 2 Departments of Internal Medicine and Metabolic-Surgical Weight Management, Kaiser Permanente, Denver, USA

**Keywords:** lifestyle modification, obesity, primary care

## Abstract

Primary care physicians can play a key role in supporting patients after behavioural weight loss, though little is known about communication between patients and physicians during this time. Adults (*n*=139) in a behavioural weight loss trial (delivered outside of primary care) who attended a primary care appointment after an initial weight loss period were surveyed to assess weight-related communication at their most recent appointment. Most participants (78%) reported discussing weight with their physician. Participants who discussed weight, compared to those who did not, lost more weight, had higher blood pressure, and were more likely to be male. Most (89%) reported that their physician was supportive of their weight loss, but only a few participants (6.9%) reported that their physician gave feedback on medical parameters. Areas for improvement identified include physicians providing universal support for modest weight changes and providing interpretation of medical measurements that changed due to weight loss.

## Background

Weight management is an important concern for many adults seen in primary care (Bray *et al*., 2013; Ard, 2015; Bernstein *et al*., 2016; Tsai *et al*., 2018b). The American College of Cardiology/American Heart Association Task Force recommends that primary care physicians (PCPs) discuss weight with all patients with a body mass index (BMI) of 30 and higher or a BMI of 25 and higher and at least one comorbidity (American College of Cardiology/American Heart Association Task Force on Practice Guidelines, Obesity Expert Panel, 2013, 2014). The extant literature demonstrates a wide variation in the number (13–66%) of these patients who have had weight-related discussions with their PCP (eg, told BMI was in overweight range, given advice to lose weight) (Sciamanna *et al*., 2000; Abid *et al*., 2005; Flocke *et al*., 2005; Ruser *et al*., 2005; Simkin-Silverman *et al*., 2005). When these communications occur, they often leave patients feeling stigmatized or misunderstood (Epstein and Ogden, 2005; Gudzune *et al*., 2014; Phelan *et al*., 2015). Much of this research focusses on interactions with patients not actively engaging in weight loss efforts, or with patient populations in which stage of change for weight loss is heterogeneous. Little is known about weight-related communication between PCPs and patients after weight loss incurred through behaviour change.

Communications that occur after a patient has successfully begun a weight loss effort may be especially important in promoting lasting behaviour change. Behavioural programs produce clinically significant weight loss by 6 months and then transition to maintenance of the behaviour changes and prevention of weight regain, known as a ‘weight loss maintenance’ period (Middleton *et al*., 2012; Dombrowski *et al*., 2014). Of note, although the main goal during weight loss maintenance is to maintain weight already lost, some individuals may continue to lose weight for a period of time (Butryn *et al*., 2018). Many biological and behavioural factors make engagement in long-term weight control (ie, weight loss efforts past 6 months) challenging, even more so than the initial period of weight loss (Butryn *et al*., 2011; Greenway, 2015). PCPs have an important role in supporting long-term adherence to behaviours that help maintain weight loss. This support can include praise or feedback about the impact weight loss has on other indicators of health (eg, blood pressure, cholesterol) (Tsai *et al*., 2018b). Despite the importance of these interactions, no study to our knowledge has examined the interactions between patients and PCPs during the weight loss maintenance period.

Thus, this study focussed on patient and PCP interactions during the ‘weight loss maintenance period’, defined as the 12 months after the participants had been enrolled in 6 months of behavioural weight loss treatment (ie months 6–18). Specifically, the study aimed to identify the frequency of weight discussions at PCP visits during weight loss maintenance, determine factors associated with discussing weight during a PCP visit, and characterize the type and amount of support or feedback provided by PCPs to this patient population.

## Methods

**Figure 1:**
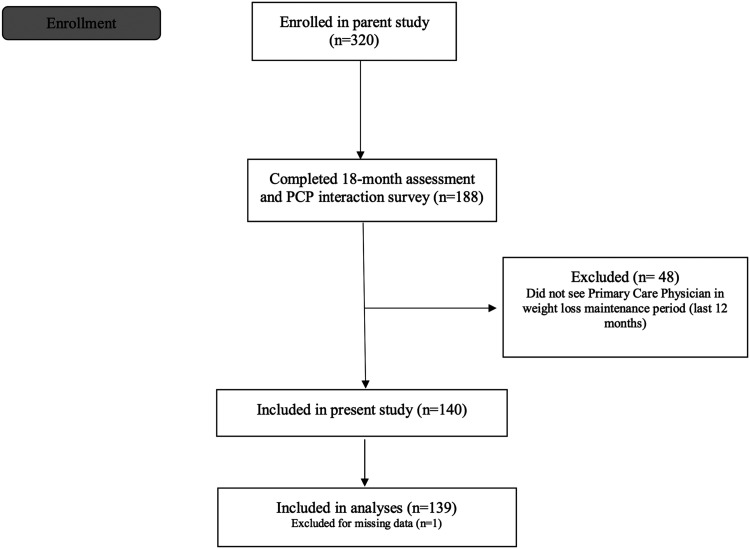
Participant flow from parent behavioural weight loss trial to current study of 139 behavioural weight loss participants who have attended an appointment with their primary care physician in the past 12 months

This is a sub-study conducted with participants originally recruited from the community for a clinical trial of group-based behavioural weight loss that provided 30 sessions of treatment over 18 months (R01DK100345; Figure 1 for participant flow; Table 1 for participant demographics). All participants were recruited from a local, urban area. Eligible participants were adults aged 18–70 years with a BMI of 27–45 kg/m^2^. Participants had to complete a 7-day food diary and be able to engage in physical activity (ie, walk at least two blocks without stopping). Individuals were ineligible if they had a medical condition that could affect participation in the study, were pregnant or planning to become pregnant, were planning to move out of the study area during the data collection period, were on medication that could cause significant change in weight, had a history of bariatric surgery, had a weight loss of ⩾5% in the previous 3 months, or were consistently engaging in ⩾150 min per week of moderate to vigorous physical activity (MVPA) in previous 6 months. To participate, patients procured their PCP’s signature on a form stating that there were no known medical contraindications for participation in the study. Otherwise, PCPs were not informed of the study and were not given guidelines on how to respond to patients. Rather, the purpose of this sub-study was to investigate the communications that occurred naturally between a PCP and patient when the patient presented after a weight loss attempt.

**Table 1:** Demographics of 139 behavioural weight loss participants

Characteristic	Measure (*n*=139)
Age in years, mean (SD)	54.9 (9.7)
Gender	
Female, *n* (%)	98 (70.5%)
Male, *n* (%)	41 (29.5%)
Race^a^	
White, *n* (%)	97 (69.8%)
Asian, *n* (%)	1 (0.7%)
Black or African American, *n* (%)	36 (25.9%)
More than one race, *n* (%)	5 (3.6%)
Percent weight loss at post-treatment (18 months), mean (SD)	11.9% (10.7%)
Months between last PCP appointment and 18-month assessment, mean (SD)	4.2 (3.2)
Reasons for last appointment with PCP	
Regular check-up, *n* (%)	103 (74.1%)
Sick/injury visit, *n* (%)	22 (15.8%)
Form signed, *n* (%)	6 (4.3%)
Other, *n* (%)	8 (5.8%)

PCP=primary care physician

a Owing to low percentage of non-White participants, race was dichotomized for analyses into participants that identified as White or non-White (ie, all other races and more than one race)

Informed consent was obtained from all participants. In the parent study participants were randomized to conditions based on the Look AHEAD and Diabetes Prevention Program Trials (two major clinical trials in the field of obesity treatment), but that varied in content (Wing *et al*., 2011; Diabetes Prevention Program Research Group, 2015). Conditions were combined for the current study because weight losses were not significantly different between conditions at 6 or 18 months (*P*<0.05), and the focus of the current study was on interactions independent of study condition.

Participants reported demographics and medical comorbidities at baseline in the weight and lifestyle inventory (Wadden and Foster, 2006). A Tanita® model WB-3000 scale, tape measure, and Omron® HEM-907 XL were used to measure participant weight, waist circumference, heart rate, and blood pressure, respectively, at baseline and 18 months. At 18 months participants responded to a self-report measure via Qualtrics, created by the investigative team, regarding their last appointment with their PCP. This measure was emailed to participants approximately 2 weeks before their in-person assessment and completed before assessment. This measure consisted of nine items that assessed the ways in which their PCP had responded to their weight loss attempt at their last appointment (Appendix 1). A subset of participants (*n*=18) elected to provide a written response summarizing what they recalled from their last appointment. No information was collected from PCPs, as this study focussed on patient perception of communication with their PCP to inform future patient-centred care.

Independent sample *t*-tests and independent χ^2^ tests were conducted to examine differences between participants who discussed weight with their PCP at their last appointment versus those that did not. Two participants had missing 6 months weight data; therefore, their closest treatment session weight was substituted for weight analyses. The pattern of analyses was the same with and without the two participants. There were minimal missing data at the item-level [*n*=6 missing data for socioeconomic status (SES) and *n*=1 missing baseline blood pressure]. These participants were excluded from the relevant statistical tests. Qualitative data provided by a subset of participants (*n*=18) were coded by the first and third author using content analysis with an inductive approach.

## Results

Participants who discussed weight with their PCPs in the past year (*n*=109) had significantly higher weight losses at both the end of the initial weight loss phase (6 months), before their appointment (mean=11.3%, SD=5.3%) and at the end of the behavioural weight loss program (18 months) after their appointment (mean=12.9%, SD=10.6%), than participants who did not discuss weight with their PCPs (*n*=30; 6 months: mean=7.9%, SD=4.9%; *P*<0.01, *d*=0.67; 18 months: mean=8.4% SD=10.7%; *P*<0.05, *d*=0.42) (Table 2). Other participant characteristics that were associated with weight discussion at a recent appointment were greater reduction in waist circumference, having higher blood pressure at 18 months, and identifying as male (all *P*s<0.05). Having a lower SES was associated with participant and PCP not discussing weight (*P*<0.05). There were no significant differences between groups in age, race, medical comorbidities at baseline of the study, baseline blood pressure, change in or 18-month resting heart rate, or change in or 18-month BMI (*P*s>0.05) (Table 2). Of note, there was a significant difference in the rates of communicating about weight versus not communicating about weight between participants whose appointments had different purposes (*χ* ^2^(3)=19.5, *P*<0.001). Participants who were scheduled for a regular check-up (eg, yearly check-up, blood pressure check) were more likely to speak with their physician about their weight (*n*=90, 82.6% communicated about weight; *n*=13, 11.9% did not). Participants who presented to their appointment for a sick/injury visit were just as likely to communicate or not communicate about weight with their physician (both *n*=11; 34.4%).

**Table 2:** Comparisons between participants discussed weight with their primary care physician and participants who did not discuss weight during weight loss maintenance in 139 behavioural weight loss participants

Measure	Discuss weight^a^ mean (SD) or *n* (%)	Did not discuss weight^b^ mean (SD) or *n* (%)	Test statistic	Significance (*P*)	Effect size (Cohen’s *d*, *w*, or *V*)
Demographics					
Age (years)	55.0 (9.5)	54.3 (10.3)	*t*(137)=0.4	0.70	0.07
Gender			*X* ^2^=(1, *n*=139)=7.0	0.008**	0.22
Male	38 (34.9%)	3 (10%)			
Female	71 (65.1%)	27 (90%)			
Race			*X* ^2^=(1, *n*=139)=1.7	0.19	0.11
White	79 (72.5%)	18 (60%)			
Non-White	30 (27.5%)	12 (40%)			
Socioeconomic status^c^			*X* ^2^=(4, *n*=134)=11.2	0.025*	0.29
$0–50,000	13 (12.5%)	4 (13.8%)			
$50,001–100,000	31 (29.5%)	17 (58.6%)			
$100,001–150,000	29 (27.6%)	6 (20.7%)			
$150,000–200,000	20 (19.0%)	2 (6.9%)			
$200,001 and up	12 (11.4%)	0 (0%)			
Health measures					
BL number of comorbidities^d^	2.9 (2.1)	2.4 (2.4)	*t*(137)=1.0	0.30	0.22
BL blood pressure^e^ ^,^ ^f^			*X* ^2^=(3, *N*=138)=0.9	0.93	0.08
Normal range	31 (28.4%)	7 (24.1%)			
Elevated range	6 (5.5%)	2 (9.9%)			
Stage 1	34 (31.2%)	10 (34.5%)			
Stage 2	36 (33%)	10 (34.5%)			
Hypertensive crisis range	2 (1.8%)	0 (0%)			
Percent weight loss BL to 6 months	11.3% (5.3)	7.9% (4.9)	*t*(137)=3.2	0.002**	0.67
Percent weight loss BL to 18 months	12.9% (10.6)	8.4% (10.7)	*t*(137)=2.0	0.045*	0.42
Change in resting heart rate BL to 18 months (beats/minute)	−4.8 (11.0)	−2.2 (13.2)	*t*(137)=−1.1	0.26	0.22
Change in waist circumference BL to 18 months (inches)	−4.2 (4.5)	−1.8 (3.6)	*t*(137)=3.7	0.008**	0.59
Change in BMI BL to 18 months	−4.6 (4.0)	−3.2 (4.4)	*t*(137)=−1.7	0.10	0.33
18 months BMI	30.1 (5.3)	31.1 (4.6)	*t*(137)=−0.9	0.35	0.20
18 months Resting heart rate (beats/minute)	69.9 (11.3)	71.4 (11.3)	*t*(137)=−0.6	0.53	0.13
18 months Blood pressure^f^			*X* ^2^=(3, *n*=139)=10.0	0.018*	0.27
Normal range	35 (32.1%)	17 (56.7%)			
Elevated range	13 (11.9%)	6 (20%)			
Stage 1	34 (31.2%)	4 (13.3%)			
Stage 2	27 (24.8%)	3 (10%)			
Hypertensive crisis range	0 (0%)	0 (0%)			

*Significance at *P*<0.05. **Significance at *P*<0.01. BL=Baseline

a *n*=109

b *n*=30

c *n*=105 discuss weight (*n*=4 missing data); *n*=29 did not discuss weight (*n*=1 missing data)

d Sum of comorbidities endorsed at baseline (heart disease, angina, palpitations, stroke, rheumatic fever, heart murmur, pacemaker, breathing problems [asthma, lung disease], high blood pressure, anaemia, back problems, joint or bone problems, hiatal hernia, arthritis, gout, gallbladder disease, thyroid problems, kidney disease, ulcers, bowel disease, liver disease, diabetes [type I or II], sleep apnoea, bodily pain, other)

e *n*=138; *n*=1 missing data

f Blood pressure categories calculated according to the American Heart Association guidelines

Most participants (89.9% of *n=*109) indicated that they received general encouragement on weight changes from their PCPs. However, only 6.9% indicated that they received feedback on medical conditions that may have improved due to their weight loss.

The qualitative results of an open-text response to what occurred in the primary care appointment when weight was discussed are displayed in Table 3 (Appendix 1). Several (*n=*4 of 18) participants reported that their PCPs were supportive and enthusiastic about changes. However, an equal number of participants (*n=*4) described their PCP as unsupportive of weight control efforts.

**Table 3:** Qualitative results from 18 behavioural weight loss participants describing their most recent weight-related communication with their primary care physician

Theme	*n*	Quotes (participant characteristics during 18-month behavioural weight loss trial)
PCP provided enthusiastic support (beyond providing general encouragement)	4	‘She is thrilled with my progress’ (Female, lost 14.7% of weight, blood pressure dropped from the elevated range to normal range over course of study, 18-month BMI of 29.3) ‘Thought I had really accomplished a great feat!’ (Female, lost 17.8% of weight, blood pressure dropped from the elevated range to normal range from baseline to 18-month, 18 months BMI of 23.50)
PCP indicated that they were unsupportive of the patient’s changes/weight loss	4	‘I brag[ged] about all the weight I lost with Project Impact (the research study), she told me I was obese.’ (Female, lost 12.3% of weight, blood pressure in Stage 1 range at baseline and 18 months, 18-month BMI of 32.7)
PCP and patient noted a gain from the last appointment	3	‘I gained weight [since my last appointment]. She encouraged me to continue with Project Impact^a^.’ (Female, lost 13.3% of weight, blood pressure at stage 1 at baseline and 18 months, 18-month BMI of 28.0)
PCP and patient discussed specific medical parameters changing	3	‘[Discussed] Cholesterol level’ (Male, lost 15% of weight, blood pressure dropped from the elevated to normal range from baseline to 18 months, 18-month BMI of 30.6)
PCP and patient discussed specific strategies to lose weight	2	‘A bit of a combination. He suggested specific diet books to read, discussed (the research study)^a^ and the main tenets of the program, suggested exercise strategies’ (Male, gained 2.6% of weight, blood pressure increased from normal to stage 1 range from baseline to 18 months, 18-month BMI of 44.8)

PCP=primary care physician; BMI=body mass index Weight and blood pressure listed above measured at 18-month study assessment, not at PCP appointment

## Discussion

This study is the first to our knowledge to explore the interactions between patients and PCPs after a period of initial weight loss. Encouragingly, most patients reported discussing weight with their PCP, and most patients indicated that their PCPs were supportive. In particular, patients with a check-up appointment (eg, yearly check-up, blood pressure check) were more likely to communicate about weight with their physician. However, few patients reported that their PCPs provided feedback on medical comorbidities, demonstrating an area for improvement. A small number of patients described that their PCPs did not understand the challenges of weight loss maintenance.

The overall pattern of effect sizes suggests that patients who discussed weight with their PCP had greater positive changes in their health than those that did not (Cohen, 1992). Patients that communicated about weight had significantly greater weight losses (12.9 versus 8.4%) and waist circumference changes. However, the group that did not discuss weight averaged approximately 8% weight loss, which is still a meaningful weight loss capable of producing improvements in quality of life and overall health (Wing *et al*., 2011; Boling and Palmer, 2015). PCPs should be aware that even small changes are difficult and should be reinforced (Wing *et al*., 2011; Jensen and Ryan, 2014), and that weight loss may be particularly difficult for patients with low self-efficacy (Pollack *et al*., 2011). Moreover, patients who lose less weight may need additional support or referrals. The 5As (ask, assess, assist, advise, and agree) for obesity management is a useful resource for weight-related communication (Osunlana *et al*., 2015). While these 5As were developed mainly for initiation of behaviour change, the structure and materials can still provide support for discussions with patients after weight loss is initiated (eg, a goal sheet, a handout on the complex processes of weight regulation) (Osunlana *et al*., 2015).

There were several demographic factors associated with patients and PCPs engaging in weight-related communication. Males were more likely than females to discuss weight with their PCP. This may be because female patients felt less comfortable initiating weight-related conversation, or because PCPs were less comfortable doing so with female patients. Several studies demonstrated that females were more likely to be diagnosed as having obesity as compared to males (McArtor *et al*., 1992; Heywood *et al*., 1996; Stafford *et al*., 2000). Additional research is needed to understand the relationship between gender and weight-related communication in primary care, particularly during weight loss maintenance. Patients with lower SES were less likely to have a weight-related conversation, perhaps because life stressors or other needs were prioritized. Previous literature demonstrates that patients’ SES negatively impacts physician–patient communication, such that patients from lower social classes receive less information and are less satisfied with communication with their physician as compared to patients from higher social classes (Willems *et al.*, 2005; Jensen *et al.*, 2010; Verlinde *et al*., 2012). PCPs should be aware of any demographic factors that may bias conversations and ensure to support healthy weight changes for all patients.

Patients with higher blood pressure at post-treatment were more likely to discuss weight with their PCP; however, a low percentage of patients reported explicitly discussing how blood pressure or other comorbidities related to their weight. Previous literature demonstrated that patients were more likely to receive advice to lose weight if they had additional weight-related comorbidities, such as hypertension (Sciamanna, 2000). In a focus group, PCPs reported utilizing communication about medical comorbidities as motivation for uptake and maintenance of weight-related changes (Gudzune *et al.*, 2012). The present study demonstrated that while patients with higher blood pressure were more likely to report discussing weight with their PCPs, only a few patients recalled that their PCPs offered specific feedback on the effects of weight loss on other measurements of health (eg, improvements in blood pressure). Therefore, recognizing the link between medical comorbidities and weight may provide a way to initiate communication about weight between PCPs and patients. However, it appears that PCPs may miss occasions to explicitly reinforce this relationship. PCP appointments are opportunities to offer feedback on physiological changes to reinforce the benefits of weight loss (Tsai *et al*., 2018b). Patients also find it motivating to see improvement in comorbidities (Ogden and Arulgnanaseelan, 2017). Measurement of comorbidities, such as blood pressure, insulin, and cholesterol levels, is important to general clinical care, and interpretation of medical comorbidities in the context of patients’ weight losses is key to support patients’ continued adherence to long-term weight control behaviours (eg, adherence to low calorie diets, engagement in regular physical activity).

When patients and PCPs discussed weight, patients described the overall communication as generally encouraging. A few patients recalled that their PCPs demonstrated a lack of support of weight control efforts. These interactions suggest that some PCPs may not understand the challenges that come with lifestyle modification in today’s obesogenic environment (Tsai *et al*., 2018a). For example, if a patient who lost over 13% of their starting weight remarks that they are experiencing some weight regain (ie, that 13% represents a weight gain from their maximum weight loss), and a PCP only encourages them to continue with their weight loss program, this suggests a lack of understanding (Table 3). If a PCP understood the challenges of weight loss maintenance, they would normalize this experience and inform the patient that their weight loss is far above average (Anderson *et al*., 2001; Butryn *et al*., 2011; Greenway, 2015). A PCP in that situation should also reinforce the immense medical benefits from a 13% weight loss, and enthusiastically support maintaining such a weight loss (Wing *et al*., 2011; Tsai *et al*., 2018b). Previous literature suggests that in addition to seeming unsupportive, PCPs can be perceived to be judgmental if they fail to acknowledge the accomplishments of their patient’s weight loss efforts, and that these negative interactions can hinder weight loss progress, and thus a patient’s health in the future (Pollack *et al*., 2010).

There are several potential limitations to note in this study. Qualitative responses were optional and provided by a small number of participants, which may bias results. This study focussed on participants who completed a behavioural weight loss trial and therefore, the results do not capture participants who dropped out of the study. Of note, the data are based solely on participants’ recall of their interaction with their physician and may be subject to patient recall bias. This study focussed on patients’ perceptions of communication to inform future patient-centred care, however, future studies may wish to collect data from PCPs to address this limitation. Other factors not measured in this study may explain aspects of weight-related interactions in primary care, such as physician perception of the interaction, PCP weight stigma, low PCP weight control knowledge, brief appointments, or PCP demographics (Yang *et al*., 2016; Tsai *et al*., 2018a). Additionally, this study focussed on patients enrolled in a research trial which may limit generalizability. Future directions include analyses that identify the most salient factors that predict frequency and quality of weight discussions during weight loss maintenance. There also needs to be additional research on the most effective communication for a PCP to deliver at this time, as little research has been conducted in this area. In particular, further research should explore SES and other demographic differences in weight-related communication (both in rates, quality, and preferences by demographic groups) to build on the findings of this study.

In summary, this study surveyed patients enrolled in a behavioural weight loss program who saw their PCPs during weight loss maintenance (ie, during months 6–18, after 6 months of successful weight loss). It is encouraging that most patients reported discussing weight with their PCP during a recent visit. Only a few patients reported receiving feedback on medical parameters, demonstrating that PCPs are missing an important opportunity to provide encouragement and motivation. Previous literature demonstrates that a lack of acknowledgement can hinger patient’s progress in health behaviour change; therefore, it is key that a PCP acknowledge as many weight-related changes as possible to further support the patient’s health (Pollack *et al*., 2010). Support can be offered through several avenues including praise for small weight decreases or weight maintenance, ongoing assessment of medical comorbidities, and additional medical aid such as intensification of behavioural treatment (eg, high-intensity counselling, meal replacement), prescription medication, or referral to bariatric surgery (Tsai *et al*., 2018b).

## Financial support

This work was supported by the National Institute of Health R01DK100345.

## Conflicts of interest

None.

## Ethical standards

The authors assert that all procedures contributing to this work comply with the ethical standards of the relevant national and Drexel University institutional guidelines on human experimentation and with the Helsinki Declaration of 1975, as revised in 2008.

## Funding information

This study was funded by National Institute of Health R01DK100345.

## Supplementary material

For supplementary material accompanying this paper visit http://dx.doi.org/10.1017/S1463423619000124. click here to view supplementary material
