# Supplementary material for: Communication between patients and primary care physicians after behavioural weight loss: an observational study
Source: Prim Health Care Res Dev. 2019 Jun 25;20:e75. doi: 10.1017/S1463423619000124 (PMC8060814; doi:10.1017/S1463423619000124)
Supplement: Supplementary file 1 [file S1463423619000124sup001.docx]

These questions are about your interactions with your primary healthcare provider around your weight. This information will **not** be shared with your healthcare provider and is **solely** for the research team to learn more about your experiences in healthcare.

1. How would you classify your primary healthcare provider?
   1. Primary Care Doctor (MD)
   2. Nurse Practitioner (NP)
   3. Physician Assistant (PA)
   4. Specialist: _____________________
   5. Other: ________________________
   6. I don’t currently have a primary healthcare provider🡪**go to question 5**
2. What year did you start seeing this primary healthcare provider?

________________

1. How often do you see this healthcare provider?
   1. Less than once a year
   2. Once a year
   3. Twice a year
   4. More than twice a Year
2. Does your doctor know you are enrolled in (the research study)?
   1. Yes
   2. No
   3. I don’t know
3. Have you seen your primary healthcare provider since you started (the research study)?
   1. Yes
   2. No**🡪 Done with questionnaire**
4. What month and year did you see your primary healthcare provider last?

______________________________

1. What was the purpose of your most recent appointment?
   1. Check-up visit (yearly check-up, blood pressure check)
   2. I was sick/injured
   3. I needed to have a form signed (i.e. insurance, work, immunizations)
   4. Other: _________________________________
2. Did you and your doctor talk about your weight at your last appointment?
   1. Yes
   2. No🡪 **Done with questionnaire**
3. What did you and your provider talk about with regards to your weight? Check all that apply.

___________Generally encouraged me to keep up my weight loss/maintenance efforts

___________ Helped me problem solve about challenges with weight control

___________Gave feedback on how my medical parameters changed since I started working on my weight (for example, if my insulin production, blood pressure, or cholesterol levels changed)

__________Other: ____________________________________________________________________________________________________________________________________________________________

______________________________________________________________________________
